# The relationship between shyness, loneliness, and mobile phone addiction in Chinese university students: a cross-lagged analysis

**DOI:** 10.3389/fpsyg.2025.1626175

**Published:** 2025-09-08

**Authors:** Jing Bai, Qi Chen, Chang Hu

**Affiliations:** ^1^Department of Leisure Sports, Kangwon National University, Samcheok-si, Republic of Korea; ^2^Faculty of Education, Languages, Psychology & Music, SEGi University, Kuala Lumpur, Malaysia; ^3^Department of Student Affairs, Changji College, Changji, Xinjiang, China; ^4^General Education Department, Wuhan College, Wuhan, Hubei, China

**Keywords:** shyness, loneliness, mobile phone addiction, longitudinal study, Chinese university students

## Abstract

**Purpose:**

Given the increasing rates of mobile phone addiction (MPA) among university students, identifying the psychological processes that contribute to this phenomenon is of vital importance for prevention and intervention. This research employs a longitudinal approach to empirically investigate the causal relationships between shyness, loneliness, and mobile phone addiction in college students.

**Methods:**

From July 2024 to March 2025, a 9-month, three-wave longitudinal survey was conducted among 404 Chinese college students using the Cheek and Buss Shyness Scale, Emotional Versus Social Loneliness Scales, and the Mobile Phone Addiction Tendency Scale.

**Results:**

(1) The synchronous and cross-lagged correlations among shyness, loneliness, and MPA were significant. (2) The direct predictive effect of shyness on MPA was found to be unstable, whereas MPA consistently predicted shyness. (3) Shyness consistently predicted loneliness, but the predictive effect of loneliness on shyness was found to be unstable. (4) Loneliness and MPA were found to be mutually predictive of each other. (5) T1 shyness significantly predicted T3 MPA through T2 loneliness; however, T3 MPA did not predict T3 shyness through T2 loneliness.

**Conclusion:**

Reducing shyness and loneliness can help alleviate MPA in college students, promoting their psychological and behavioral health. MPA may exacerbate loneliness and shyness, and early identification and intervention can help break this vicious cycle.

## 1 Introduction

With the rapid advancement of technology and ongoing innovations in smart technologies, China recorded approximately 1.091 billion mobile internet users by December 2023 (China Internet Network Information Center, 2024). Although mobile phones have substantially enhanced convenience in daily life, the excessive use of these devices has emerged as an increasingly critical global public health concern. MPA is defined as an individual’s inability to control their excessive and frequent use of mobile phones (Kwon et al., 2013). In recent years, MPA has become particularly prominent among Chinese university students, which is closely related to their unique social context. Most Chinese university students study away from home and live apart from their families, resulting in relatively loose parental supervision. Additionally, college students in China typically face less academic workload and pressure, and have ample discretionary time, creating favorable conditions for frequent smartphone use and thus increasing the risk of addiction (Chi et al., 2016). Meanwhile, the negative impacts of MPA have become increasingly prominent, with existing research confirming its detrimental effects on college students’ physical health, mental health, and sleep quality (Mei et al., 2023). Consequently, identifying the potential risk factors associated with MPA and elucidating its underlying mechanisms is of considerable theoretical and practical significance for its prevention and intervention.

Extant studies have identified shyness and loneliness as two pivotal psychological predictors of MPA (Bian and Leung, 2015). Shy individuals, in order to cope with discomfort experienced in social situations, are inclined to use mobile phones as an escape from face-to-face interactions, thereby increasing the risk of developing MPA (Bian and Leung, 2015; Enez Darcin et al., 2016). Similarly, individuals with elevated levels of loneliness may gravitate toward the virtual world to compensate for the absence of connection and support in their offline social lives (Yao and Zhong, 2014). Furthermore, research suggests that shyness may indirectly affect MPA through loneliness, with loneliness as a mediating variable between the two (Aktaş and Yılmaz, 2017). Individuals with shyness tend to withdraw from and avoid real-life social interactions, making it difficult for them to obtain adequate social support. As a result, they are more likely to experience elevated levels of loneliness, which further drive them to use their smartphones more frequently in search of virtual belonging, thereby increasing the risk of MPA. On the other hand, MPA may also undermine individuals’ real-life social skills and reduce their self-efficacy, leading to greater feelings of loneliness and social difficulties, which further exacerbate shyness (Tian et al., 2018).

Although previous studies have elucidated the relationships among shyness, loneliness, and MPA, most of this research has adopted cross-sectional designs, relying on data collected simultaneously to infer associations among variables. Such approaches are limited in their ability to accurately reveal the causal ordering and dynamic developmental processes among shyness, loneliness, and MPA. Therefore, based on the existing research, the present study employs a longitudinal cross-lagged design to systematically examine the influence mechanisms of individual trait factors (shyness) and psychological states (loneliness) on MPA. This study aims to uncover the dynamic interactive relationships and potential mediating pathways among these three variables. By conducting an in-depth analysis of the causal pathways among variables, this research seeks to advance the understanding of the mechanisms through which shyness and loneliness contribute to the development of MPA, thereby providing theoretical foundations and practical guidance for targeted psychological interventions and behavioral correction programs in higher education settings.

### 1.1 Association between shyness and MPA

Shyness, a distinct and profound personality trait, is primarily characterized by tension, apprehension, and discomfort experienced in social situations (Zimbardo, 1977). Cognitively, shyness manifests as heightened sensitivity and an excessive concern with others’ perceptions and attitudes; emotionally, it is associated with a fear of negative evaluation by others, often leading to anxiety, unease, and other negative emotional states (Crazier, 1979); behaviorally, it is reflected in social inhibition and the avoidance or withdrawal from social interactions. In recent years, with the widespread adoption and advancement of mobile phones, individuals with high levels of shyness have increasingly turned to mobile phones as a means of alternative social engagement when confronted with social pressures, drawing scholarly attention to the relationship between shyness and MPA.

Although prior work has largely examined the link between shyness and MPA, the majority of investigations have relied on one-way path analysis, either examining the influence of shyness on MPA (Chen et al., 2021; Li et al., 2022) or the effect of MPA on shyness (Eroğlu et al., 2013). However, such unidirectional causal assumptions may overlook the potential dynamic and reciprocal interactions between these two variables. Theoretically, shyness may lead individuals to engage more frequently with mobile phones as a strategy to avoid real-world social interactions, thereby increasing the risk of addiction. Conversely, MPA, by impairing social skills and further promoting avoidance of face-to-face communication, may, in turn, exacerbate levels of shyness. Therefore, the relationship between shyness and MPA is likely to be bidirectional and mutually reinforcing. Accordingly, Hypothesis 1 is proposed: Shyness and MPA are dynamically and bidirectionally associated.

### 1.2 Association between shyness and loneliness

Numerous studies have indicated that individuals with high levels of shyness often exhibit greater social anxiety and lower self-esteem in social interactions (Cowden, 2005; İme et al., 2024) and frequently encounter difficulties in establishing and maintaining interpersonal relationships. Among the various adverse outcomes associated with shyness, loneliness is among the most prevalent (Ashe and McCutcheon, 2001; Jackson et al., 2002). Loneliness is an emotional experience resulting from the unmet needs for intimacy and social connection (Russell et al., 1980). This negative emotional state emerges when there is a marked gap between the social connections an individual hopes to have and those they actually experience, in terms of both quality and quantity.

In recent years, scholars have explored how shyness relates to loneliness, with research indicating that people who exhibit greater shyness are more prone to experiencing heightened feelings of loneliness (Jackson et al., 2002; Wang et al., 2020). Shy individuals, often due to a lack of self-confidence and social skills (Crazier, 1979), frequently display behaviors such as withdrawal, hesitation, and self-deprecation during face-to-face interactions. This tendency toward social avoidance significantly reduces opportunities for establishing deep interpersonal connections, intensifying feelings of loneliness.

Although shyness is typically regarded as an antecedent of loneliness, some studies have suggested that loneliness may, in turn, influence levels of shyness. Prolonged experiences of loneliness can undermine individuals’ social self-efficacy and self-confidence (Al Khatib, 2012), leading to increased negative expectations in social situations, such as fears of rejection or negative evaluation. These anxieties manifest behaviorally as social avoidance and withdrawal, hallmark characteristics of shyness. Therefore, loneliness may not only be an outcome of shyness but also contribute to its development. Accordingly, Hypothesis 2 is proposed: Shyness and loneliness are dynamically and bidirectionally associated.

### 1.3 Association between loneliness and MPA

Loneliness affects individuals’ emotional states and profoundly impacts their daily behaviors, particularly mobile phone use. Research has shown that loneliness positively predicts MPA (Dayapoglu et al., 2016). Individuals with high levels of loneliness, who often find it difficult to obtain sufficient social support and close relationships in real life, tend to seek emotional compensation and social connection through mobile phone social applications. Such compensatory use significantly increases the risk of developing MPA (O’Day and Heimberg, 2021). In addition, to alleviate the negative emotions associated with loneliness, lonely individuals are often drawn to entertainment activities such as online gaming, online shopping, and watching short videos (Luo et al., 2022; Kwon et al., 2011; Whitty and McLaughlin, 2007; Zhao and Kou, 2024). Although college students may initially use mobile phones to mitigate negative emotions, seek social support, or pursue entertainment, a lack of proper control and intervention over such usage patterns can progressively lead to the development of MPA.

However, MPA may also predict loneliness (Su and He, 2024). On one hand, excessive reliance on the internet or mobile phones may impair individuals’ social adaptation abilities (Zhao et al., 2022), resulting in reduced perceived social support in real-world settings and thereby intensifying feelings of loneliness. On the other hand, the instant gratification derived from immersion in virtual environments may lead individuals to avoid real-life interpersonal interactions, further exacerbating their sense of social isolation. Therefore, loneliness may serve not only as a precursor to MPA but also as one of its consequences. Accordingly, Hypothesis 3 is proposed: Loneliness and MPA are dynamically and bidirectionally associated.

### 1.4 Mediating role of loneliness in the association between shyness and MPA

Individuals with shyness tend to avoid face-to-face social interactions due to concerns about social failure or negative evaluation, which leads to greater difficulties and challenges in interpersonal relationships. Such barriers in real-life social contexts often hinder them from obtaining adequate social support, thereby increasing their experience of loneliness (Zhao et al., 2013). In the digital age, mobile phones offer shy college students a low-pressure and highly controllable channel for online socialization, enabling them to express themselves more freely and establish virtual connections, thus partially compensating for the lack of real-life social interactions (Elhai et al., 2017). In addition, the abundant information resources and entertainment content available on smartphones can help distract attention and alleviate the negative emotions caused by social anxiety or loneliness (Zhou and Shen, 2024). However, long-term reliance on such online interaction patterns, although able to temporarily relieve emotional loneliness and stress, may also weaken real-world social skills, reinforce emotional dependence on mobile phones, and increase the risk of MPA (Zhang et al., 2023). Existing studies have indicated that loneliness is not only a key factor driving shy individuals to use mobile phones, but may also serve as a mediator in the relationship between shyness and MPA; that is, shyness increases individuals’ dependence on mobile phones by heightening their sense of loneliness. Based on the above theoretical and empirical foundations, Hypothesis 4 is proposed: Loneliness significantly mediates the longitudinal pathway from shyness to MPA.

Conversely, loneliness may also mediate the influence of MPA on shyness. Specifically, individuals with MPA tend to devote substantial time and energy to virtual social activities, which often reduces opportunities for real-world social engagement (Kuss and Griffiths, 2011). This substitution may increase discomfort and anxiety in in-person interactions, thereby triggering social avoidance behaviors that eventually intensify feelings of loneliness and shyness. Accordingly, Hypothesis 5 is proposed: Loneliness significantly mediates the longitudinal pathway from MPA to shyness.

## 2 Materials and methods

### 2.1 Participants and procedures

The present study was approved by the Ethics Committee of ChangJi College. Before the formal survey, the study’s purpose and procedures were explained to the schools, teachers, and participants, and informed consent was obtained. Participants were informed that they could voluntarily continue participating or withdraw from the study at any stage. Data were collected through paper-based questionnaires administered during class sessions beginning in July 2024. The longitudinal tracking was conducted over 9 months across three time points: July 2024 (T1), November 2024 (T2), and March 2025 (T3). Initially, a total of 546 college students participated in the study. After excluding 142 students who withdrew midway or provided incomplete responses, the final sample comprised 404 students (127 males and 277 females).

### 2.2 Measures

#### 2.2.1 Cheek and buss shyness scale (CBSS)

Shyness was assessed using the CBSS, originally developed by Cheek and Buss and later adapted for Chinese university students by Xiang et al. (2018). This unidimensional scale comprises 13 items and employs a five-point Likert response format, with higher scores indicating greater shyness. Sample items for this scale include: “I often feel uncomfortable at parties or other social gatherings”; “It takes me a long time to overcome my shyness in new situations.” The Chinese version has demonstrated satisfactory psychometric properties in previous studies (Zhao et al., 2012, 2013). The Cronbach’s α values for shyness across three time points were 0.898, 0.884, and 0.887.

#### 2.2.2 Emotional versus social loneliness scales (ESLS)

Loneliness was assessed using the ESLS, developed by Wittenberg based on Weiss’s theoretical framework and revised by Wang et al. (1999) for use in Chinese samples. This scale comprises 10 items and adopts a five-point Likert response format. Sample items for this scale include: “I feel that I do not really belong to my group of friends”; “No one maintains a special relationship with me that makes me feel understood.” The Chinese version of the ESLS has shown satisfactory reliability and validity (Kong and You, 2013). The Cronbach’s α values for loneliness across three time points were 0.873, 0.901, and 0.904.

#### 2.2.3 Mobile phone addiction tendency scale (MPATS)

Mobile phone addiction was assessed using the MPATS developed by Xiong et al. (2012) to evaluate MPA tendencies among college students. This scale comprises 16 items and adopts a five-point Likert response format. Sample items for this scale include: “I feel uncomfortable if I am without my mobile phone for a long period of time”; “My mobile phone feels like a part of me; without it, I feel as if something is missing.” The Chinese version of the MPATS has demonstrated satisfactory reliability and validity (Xiong et al., 2012). The Cronbach’s α values for MPA across three time points were 0.875, 0.871, and 0.871.

### 2.4 Data analysis

Data were analyzed using SPSS 25.0 and AMOS 24.0. The specific analytical procedures were as follows: First, SPSS 25.0 was used to conduct reliability analysis, common method bias testing, and correlation analysis for each study variable, in order to explore the basic relationships and internal associations among the variables. Subsequently, a cross-lagged panel model was constructed using AMOS 24.0, and maximum likelihood estimation was employed to examine the cross-lagged effects among the variables, thereby verifying the dynamic causal relationships. To further assess the indirect effects between variables within the model, a bootstrap analysis was conducted to ensure the reliability and statistical significance of the mediation effects.

## 3 Results

### 3.1 Common method bias test

The Harman single-factor test was conducted to examine common method bias. The results indicated that the number of factors with eigenvalues greater than 1 in the three rounds of testing were 6, 7, and 7, respectively. The percentage of variance explained by the first unrotated factor in each round was 22.51, 21.87, and 20.02%, all below the critical threshold of 40%. Thus, no common method bias was found in any of the three measurements of this study.

### 3.2 Descriptive and correlation analysis

Descriptive statistics and correlation analyses were performed for all variables, and the results are presented in Table 1. (1) Stability of Correlations: The correlations of shyness across the three testing points were significant and positive: T1 shyness with T2 shyness (*r* = 0.28), T1 shyness with T3 shyness (*r* = 0.19), and T2 shyness with T3 shyness (*r* = 0.30), all significant at *P* < 0.001. The correlations of loneliness across the three testing points were also significant and positive: T1 loneliness with T2 loneliness (*r* = 0.32), T1 loneliness with T3 loneliness (*r* = 0.17), and T2 loneliness with T3 loneliness (*r* = 0.28), all significant at *P* < 0.001. Regarding MPA, the correlations across the three testing points were significant and positive: T1 MPA with T2 MPA (*r* = 0.30), T1 MPA with T3 MPA (*r* = 0.38), and T2 MPA with T3 MPA (*r* = 0.23), all significant at *P* < 0.001. (2) Synchronicity of Correlations: In the first round of testing, T1 shyness, T1 loneliness, and T1 MPA were significantly correlated with each other (*P* < 0.001). In the second round, T2 shyness, T2 loneliness, and T2 MPA were significantly correlated (*P* < 0.001). In the third round, T3 shyness, T3 loneliness, and T3 MPA were significantly correlated (*P* < 0.001) (see Table 1). These results suggest that shyness, loneliness, and MPA exhibit cross-time stability and synchronicity of correlations, making them suitable for cross-lagged analysis.

**TABLE 1:** Descriptive statistics and correlation analysis of all variables (*n* = 404).

	1	2	3	4	5	6	7	8	9
1. T1 shyness	1								
2. T1 loneliness	0.29^***^	1							
3. T1 MPA	0.29^***^	0.34^***^	1						
4. T2 shyness	0.28^***^	0.29^***^	0.41^***^	1					
5. T2 loneliness	0.28^***^	0.32^***^	0.44^***^	0.29^***^	1				
6.T2 MPA	0.15^***^	0.28^***^	0.30^***^	0.24^***^	0.25^***^	1			
7.T3 shyness	0.19^***^	0.23^***^	0.30^***^	0.30^***^	0.17^***^	0.22^***^	1		
8.T3 loneliness	0.17^***^	0.17^***^	0.23^***^	0.23^***^	0.28^***^	0.21^***^	0.17^***^	1	
9.T3 MPA	0.27^***^	0.27^***^	0.38^***^	0.31^***^	0.32^***^	0.23^***^	0.21^***^	0.25^***^	1
M	2.75	1.98	2.49	2.64	1.89	2.80	2.74	2.11	3.05
SD	0.69	0.66	0.52	0.84	0.77	0.50	0.79	0.77	0.54

***P < 0.001; T1, T2, and T3 represent Time Point 1, Time Point 2, and Time Point 3, respectively. Column numbers (1–9) correspond to the same variables as those indicated in the row headings.

### 3.3 Cross-lagged analysis of shyness, loneliness, and MPA in college students

**FIGURE 1:**
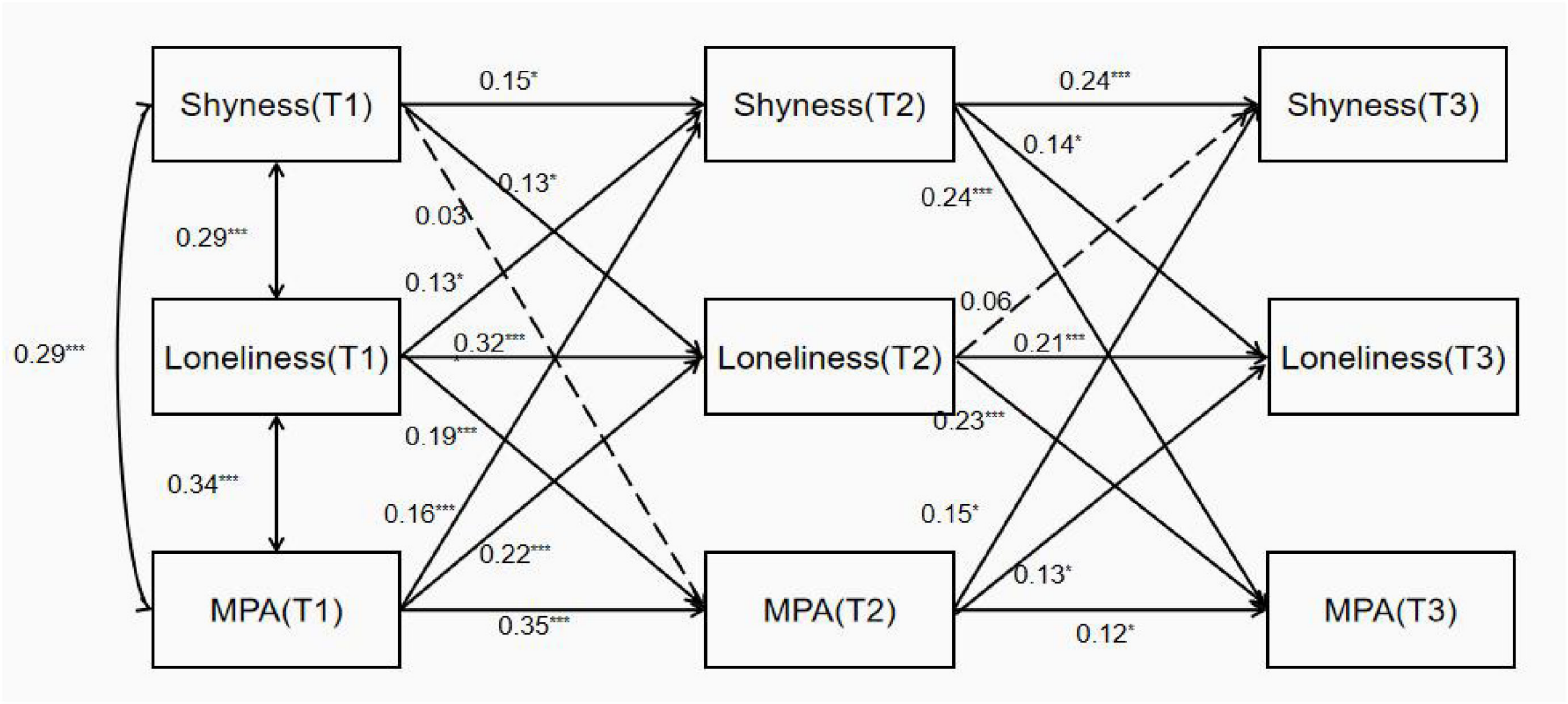
Standardized structural model of longitudinal cross-lagged panel analysis. *P < 0.05, **P < 0.01, ***P < 0.001. Solid lines represent significant relationships, while dashed lines represent non-significant relationships.

A cross-lagged effects model was constructed using AMOS 24.0 software, and maximum likelihood estimation was employed to examine the model fit. The model fit indices revealed: χ^2^ = 61.15, df = 15, χ^2^/df = 4.08, *P* < 0.001; goodness-of-fit indices: GFI = 0.96, AGFI = 0.89, CFI = 0.92, ILI = 0.92; root mean square error of approximation (RMSEA) = 0.087. These model fit indices confirm that the constructed cross-lagged effects model demonstrates good fit and adaptability (Hou et al., 2004). Using the path coefficients from the cross-lagged effects model, we investigated the asynchronous relationships between shyness, loneliness, and adolescent MPA. (1) T1 shyness had a significant effect on T2 loneliness (β = 0.13, *P* < 0.05), but no significant effect on T2 MPA (β = 0.03, *P* > 0.05). T1 loneliness significantly influenced T2 shyness (β = 0.13, *P* < 0.05) and T2 MPA (β = 0.19, *P* < 0.001). T1 MPA significantly affected T2 shyness (β = 0.16, *P* < 0.001) and T2 loneliness (β = 0.22, *P* < 0.001). (2) T2 shyness significantly influenced T3 loneliness (β = 0.14, *P* < 0.01) and T3 MPA (β = 0.24, *P* < 0.001). T2 loneliness significantly influenced T3 MPA (β = 0.23, *P* < 0.001), but had no significant effect on T3 shyness (β = 0.06, *P* > 0.05). T2 MPA had no significant effect on T3 shyness (β = 0.15, *P* > 0.05) or T3 loneliness (β = 0.13, *P* > 0.05) (Figure 1). Following the methodology used in previous studies that analyzed causal relationships between variables with cross-lagged models, and based on the path coefficients, it can be concluded that shyness and loneliness are antecedent factors of MPA in college students. Furthermore, shyness also serves as a precursor to loneliness. From the temporal sequence of relationships between variables, loneliness plays a mediating role in the pathway from shyness to adolescent MPA. Bootstrap analysis showed that T2 loneliness (ab = 0.058, *P* = 0.001, 95% CI = [0.035, 0.090]) had a significant indirect effect in predicting T3 MPA by T1 shyness.

## 4 Discussion

### 4.1 Reciprocal relationship between shyness and MPA

This study utilized a cross-lagged panel model to examine the longitudinal relationship between shyness and MPA among Chinese college students. Findings indicated a reciprocal and evolving link between the two variables. However, the predictive effect of shyness on MPA was inconsistent across time points; specifically, shyness at T1 did not significantly predict MPA at T2, whereas shyness at T2 did predict MPA at T3. These findings suggest that shyness may, to some extent, predict tendencies toward MPA among college students. One possible explanation is that shy individuals typically have smaller social networks, engage in fewer social interactions, and experience lower levels of social support (Crozier, 2002; Lazarus, 1982). According to cognitive-behavioral theory, behavioral patterns are influenced by internal cognitive frameworks. Shy individuals often exhibit cognitive biases, such as negative self-perceptions regarding their social competence or exaggerated perceptions of threats in social contexts, contributing to social avoidance behaviors. The anonymity, convenience, and escapism afforded by the Internet and mobile phones make these technologies particularly attractive to shy individuals (McKenna and Bargh, 1999). In contrast to face-to-face conversations, online communication affords individuals more control over response timing, allowing thoughtful reflection and message refinement. This temporal flexibility can meaningfully reduce social pressure. However, over time, such avoidance-based coping strategies may evolve into dependence, eventually leading to MPA. Although there is an association between shyness and MPA, the predictive relationship is not consistently stable. Multiple mediating factors may moderate this instability. Previous research has highlighted the mediating roles of social anxiety and loneliness (Bian and Leung, 2015; Li et al., 2022). Self-regulation and the satisfaction of basic psychological needs have been identified as pivotal mediators in the link between shyness and MPA (Chen et al., 2021; Sun and Yu, 2024). Therefore, the influence of shyness on MPA is not a simple causal relationship but rather a complex process regulated by multiple interacting psychological mechanisms.

On the other hand, MPA is not merely a potential outcome of shyness; it may also act as an antecedent. Specifically, MPA at T1 significantly predicted shyness at T2, and MPA at T2 significantly predicted shyness at T3, consistent with previous research findings (Jiang et al., 2023). College students with MPA tend to immerse themselves in virtual social environments for extended periods, resulting in a significant reduction in real-world social engagement, which restricts the development of social skills (Tetik et al., 2025). Deficits in interpersonal competence may, in turn, trigger heightened unease and tension during in-person interactions, thereby reinforcing shy behavior. Furthermore, individuals accustomed to expressing themselves and interacting through mobile phones may experience difficulties adapting to in-person communication. In summary, the relationship between shyness and MPA is not unidirectional but rather a bidirectional, dynamic, and evolving process, shaped by the interplay of multiple psychological mechanisms and behavioral pathways. Thus, Hypothesis 1 was partially supported.

### 4.2 Reciprocal relationship between shyness and loneliness

This study demonstrated a mutual influence between shyness and loneliness. Over time, higher levels of shyness consistently contributed to increased feelings of loneliness among college students. Specifically, shyness at T1 predicted loneliness at T2, and shyness at T2 predicted loneliness at T3, consistent with previous research findings (Davis, 2001). At the behavioral level, shy individuals tend to withdraw from social engagement as a result of low self-confidence in real-life social situations (Crozier, 1990). The college years are a critical period for individual socialization, during which it is essential to actively build social networks to adapt to broader social development. However, the social withdrawal exhibited by shy college students impedes this process, thereby intensifying experiences of loneliness. At the cognitive level, shy students often negatively evaluate their social competence and display cognitive biases during social information processing. In particular, when faced with ambiguous or neutral social cues, they are prone to negative attributions, such as interpreting others’ silence or indifference as rejection or disapproval (Blöte et al., 2019). Such negative self-evaluation and biased interpretation of others’ intentions not only heighten individuals’ feelings of insecurity and anxiety in social situations, but also lead them to avoid interactions or remain silent as a means of preventing potential negative outcomes (Alden and Taylor, 2004). This persistent avoidance behavior inadvertently reduces opportunities for social support and positive interactions, thereby exacerbating feelings of loneliness.

When examining the inverse effect of loneliness on shyness, the results indicate that its predictive effect exhibits both stage-specificity and instability. Specifically, loneliness at T1 significantly predicted shyness at T2, whereas loneliness at T2 did not significantly predict shyness at T3. Although loneliness can exacerbate social anxiety and avoidance behaviors in the short term, thereby increasing shyness (Tian et al., 2018), in the long term, loneliness primarily reflects individuals’ subjective experience of lacking social connections, whereas shyness is more related to self-evaluations of social competence and concerns about negative evaluation in social situations. More specifically, loneliness often motivates individuals to actively seek social support to alleviate feelings of inner emptiness, whereas shy individuals tend to avoid social interactions due to concerns about social failure or negative evaluation (Ashe and McCutcheon, 2001; Perlman and Peplau, 1981). Therefore, the influence of loneliness on shyness is more likely to be characterized by stage-specific and unstable dynamics, and Hypothesis 2 in this study was only partially supported.

### 4.3 Reciprocal relationship between loneliness and MPA

The findings indicated a reciprocal, time-lagged association between loneliness and MPA. Loneliness significantly and positively predicted subsequent stages of MPA among college students over time. Specifically, loneliness at T1 predicted MPA at T2, and loneliness at T2 predicted MPA at T3, consistent with previous research findings (Tian et al., 2018). According to self-determination theory, individuals possess three basic psychological needs: autonomy, competence, and relatedness. Loneliness, as a manifestation of unmet relatedness needs, drives college students to seek alternative mechanisms for social compensation. In this process, mobile phones serve as a substitute for real-world social interaction, allowing individuals to obtain social support through virtual activities such as liking, commenting, and messaging, thereby alleviating feelings of loneliness. Additionally, from an emotion regulation standpoint, loneliness is frequently linked to adverse emotional states, including depression, anxiety, and a sense of emptiness (Cacioppo and Cacioppo, 2018; Elhai et al., 2017). Due to their convenience and immediacy, mobile phones provide a wide range of online activities, such as entertainment browsing and online gaming, which can quickly fulfill the emotional needs of lonely individuals and temporarily relieve negative emotions. This immediate positive reinforcement gradually strengthens an internal reliance on mobile phones as the primary tool for emotional management, ultimately leading to the development of MPA.

Mobile phone addiction was also found to significantly and positively predict loneliness over time. Specifically, MPA at T1 predicted loneliness at T2, and MPA at T2 predicted loneliness at T3. Excessive use of mobile phones reduces actual interactions with friends and family, weakening face-to-face communication skills and diminishing the quality of real-world interpersonal relationships, ultimately increasing feelings of loneliness (Twenge et al., 2019). Thus, loneliness and MPA form a mutually reinforcing, vicious cycle: increased loneliness prompts higher mobile phone use in an attempt to seek emotional compensation, whereas excessive reliance on mobile phones further reduces real-world social activities, leading to lower frequency and quality of face-to-face interactions and consequently exacerbating loneliness. Therefore, Hypothesis 3 was supported.

### 4.4 Mediating role of loneliness in the influence of shyness on MPA

The results of this study indicate that loneliness functions as a significant mediator in the longitudinal pathway from shyness to MPA. Specifically, shyness at T1 predicts MPA at T3 through the mediating effect of loneliness at T2. From a theoretical standpoint, the Interaction of Person-Affect-Cognition-Execution (I-PACE) model offers a compelling framework for interpreting this pathway. According to the I-PACE model, the development of behavioral addictions results from the interaction of multiple factors, including personal characteristics, affective experiences, cognitive responses, and executive control (Brand et al., 2019, 2025). In the context of the present study, shyness, as a salient individual trait, tends to lead to social withdrawal and avoidance in real-life social situations, thereby making it challenging for individuals to obtain sufficient social support and consequently increasing their susceptibility to loneliness (Zhao et al., 2013). Consistent with the I-PACE model, loneliness, as a negative emotional state, may drive individuals to seek emotional compensation and social connection via digital media, such as virtual social networking, short videos, and online entertainment, in an effort to alleviate inner loneliness and distress. However, prolonged reliance on mobile phones as the principal means of emotional regulation and gratification can undermine self-control and executive function, leading to increasingly automatic and compulsive patterns of use, and thereby substantially elevating the risk of MPA. Therefore, loneliness serves as a crucial bridge in the relationship between shyness and MPA, and the present findings provide empirical support for Hypothesis 4.

However, loneliness did not exhibit a significant mediating effect in the cross-lagged pathway from MPA to shyness. Specifically, although MPA at T1 significantly predicted loneliness at T2, loneliness at T2 did not subsequently predict shyness at T3. Excessive use of mobile phones reduces real-life social interactions, thereby exacerbating individuals’ experiences of loneliness (Su and He, 2024); nevertheless, this loneliness does not appear to be sufficient to further elevate levels of shyness. Under the influence of MPA, individuals’ social skills are often impaired. This decline in social competence is more likely to directly contribute to increased shyness, rather than operating through the indirect pathway of loneliness. In other words, the negative impact of MPA on the development of shyness is primarily exerted through direct mechanisms, and the mediating role of loneliness is relatively limited. Therefore, Hypothesis 5 was not supported in the present study.

## 5 Conclusion

Using cross-lagged regression analysis, this study examined the causal and developmental relationships among shyness, loneliness, and MPA in college students. The results indicated that: (1) The synchronous and cross-lagged correlations among shyness, loneliness, and MPA were significant. (2) The direct predictive effect of shyness on MPA was found to be unstable, whereas MPA consistently predicted shyness. (3) Shyness consistently predicted loneliness, but the predictive effect of loneliness on shyness was found to be unstable. (4) Loneliness and MPA were found to be mutually predictive of each other. (5) T1 shyness significantly predicted T3 MPA through T2 loneliness; however, T3 MPA did not predict T3 shyness through T2 loneliness.

The results offer valuable insights into the psychological and behavioral well-being of college students: (1) In addressing MPA among college students, it is crucial not only to focus on correcting superficial behaviors but also to pay deeper attention to emotional experiences and personality traits, particularly the social barriers and negative emotional experiences associated with shyness. Interventions targeting shy students should include social skills training and emotional regulation programs to enhance self-confidence and social self-efficacy, thereby alleviating loneliness and reducing the motivation to seek compensatory satisfaction through mobile phone use, and ultimately promoting self-regulation of mobile phone behavior. (2) The reverse predictive effect of MPA on loneliness and shyness suggests that MPA is not merely a consequence of psychological issues but may also serve as a new risk factor that exacerbates negative emotional experiences and social difficulties. Therefore, early identification and intervention for students with signs of MPA are essential. Strategies such as cognitive-behavioral therapy and time management training can help students use mobile phones more rationally and break the vicious cycle of MPA, loneliness, and shyness.

## 6 Limitations and future directions

Although this study employed a longitudinal design and cross-lagged modeling to explore the dynamic relationships among shyness, loneliness, and MPA, several limitations remain, warranting further refinement in future research. First, the data were obtained via self-reported questionnaires, and the participants were mainly university students from Shanxi Province, which may restrict the broader applicability of the results. Future studies are encouraged to include more diverse samples across various age ranges, cultural contexts, and professional groups to improve the generalizability of the conclusions. Second, besides shyness and loneliness, other factors (e.g., stress, social support, and self-regulation) were not included in this study’s analysis. Future research could further clarify the influence of these psychological factors on MPA.

## Data Availability

The raw data supporting the conclusions of this article will be made available by the authors, without undue reservation.
